# The Refinement of Home Exercise Program for Children and Adolescents With Muscular Dystrophy in the Present COVID-19 Pandemic Scenario: A Scoping Review

**DOI:** 10.7759/cureus.29344

**Published:** 2022-09-19

**Authors:** Pallavi Harjpal, Rakesh K Kovela, Anushka Raipure, Charul Dandale, Moh'd Irshad Qureshi

**Affiliations:** 1 Physiotherapy, Ravi Nair Physiotherapy College, Datta Meghe Institute of Medical Sciences, Wardha, IND; 2 Physiotherapy, Nitte Institute of Physiotherapy, NITTE (Deemed to be University), Mangalore, IND; 3 Neuro-Physiotherapy, Ravi Nair Physiotherapy College, Datta Meghe Institute of Medical Sciences, Wardha, IND

**Keywords:** physical activity, rehabilitation, physiotherapy, covid-19 retro, home exercise program, muscular dystrophy

## Abstract

Muscular dystrophies (MDs) are a category of hereditary illnesses characterized by the gradual malfunction and/or weakening of the skeletal muscles. This disease of the muscles also results in hypotonia and joint contracture, along with raised serum creatine kinase (CK) levels. To prevent complications, continuous physiotherapy is advised for children with muscular dystrophy, which is even asked to perform at home as a home exercise program (HEP). As a result, the home exercise program (HEP) is critical in maintaining the optimal health of children with Duchenne muscular dystrophy (DMD). The present coronavirus (COVID-19) pandemic has adversely affected these children as there was very little scope to get direct help from a physiotherapist. Meanwhile, the home program was continued by many to compensate for the direct benefit. However, because of the lack of specific guidelines and structured methodology to follow for a home program, there was a deterioration in the health status of many children. There is a need to understand how the children are getting affected and the way the home program can be refined to help needy children with muscular dystrophy. Our scoping review aims to identify the present home program patterns being followed for children with DMD and their scope for refinement. The data were collected from electronic databases including PubMed, ProQuest, Cochrane, and Web of Science. We searched four electronic databases until September 2021. We included the published case studies, observational and experimental studies that described the positive impact of home exercise programs, and the methodology they followed as an alternative to institution-based physiotherapy. One hundred thirty-eight titles were screened, and 58 met the inclusion criteria. Along with regular physiotherapy, the incorporation of HEP helped in early complication prevention in patients with muscular dystrophy. The HEP was found to be a successful adjunct in the COVID-19 scenario. This review presents different therapeutic measures that can be taken for the prevention of complications in patients with MD and how the HEP plays an important role in removing the gaps on how HEP is beneficial in the COVID-19 scenario and a scope to refine the present methodologies for more accurate management.

## Introduction and background

Muscular dystrophy

Muscular dystrophy (MD) primarily affects the muscles. They are genetically inherited disorders, which are typically degenerative [1]. It's a cluster of over 30 genetic diseases marked by muscle weakness and the death of muscle cells, which are replaced by connective tissue and adipose tissue [2]. Muscular dystrophies result in progressive muscle weakness, hypotonia, and joint contractures, along with raised serum creatine kinase (CK) levels [3]. Because some varieties of muscular dystrophy are more frequent than others, the overall incidence of muscular dystrophy varies [4]. Duchenne muscular dystrophy (DMD), Becker muscular dystrophy (BMD), Emery-Dreifuss muscular dystrophy (EDMD), facioscapulohumeral muscular dystrophy (FSHD), and congenital muscular dystrophy (CMD) are the various types [1].

The most common form is Duchenne muscular dystrophy (DMD), which occurs in one in every 3,600 male live births [5]. The cause of DMD is mutations in the dystrophin gene on the X chromosome (at Xp21.1) [2]. Gross motor delay or an atypical gait is the most common presenting symptom [6]. The lower extremities are affected first by the proximal weakness, which progresses to the point where wheelchair use is essential. When patients sit, they have calf hypertrophy and lumbar lordosis that disappears. Glucocorticoids are a recommended treatment in DMD and have been linked to increased ambulatory capability, enhanced respiratory function, and increased upper limb strength [7]. The initiation of glucocorticoid medication, before the loss of ambulation, is advantageous [8].

Becker muscular dystrophy (BMD) is a type of muscle dystrophin deficiency caused by a mutation in the DMD gene. Even though BMD has a less severe clinical history than Duchenne muscular dystrophy (DMD), the phenotypic heterogeneity is great [9]. Facial muscles, shoulder girdles, and upper arms are affected by facioscapulohumeral muscular dystrophy (FSHD), which is followed by trunk and distal lower extremities, and more proximal muscular weakening later in the disease course. There is considerable variation in presentation and progression rates [10].

Congenital muscle dystrophies (CMDs) are a rare group of MD illnesses characterized by the beginning of muscle weakness before the age of one year, appearing as more or less severe newborn hypotonia and histologically presenting with dystrophic lesions [11]. Congenital dystrophies are present at birth [8].

Emery-Dreifuss muscular dystrophy (EDMD) is a rare MD with serious cardiac complications. It's divided into three types based on how it's passed down through the generations: X-linked, autosomal dominant, and autosomal recessive. All three are differentiated by a clinical triad of joint abnormalities that begin in childhood and progress to muscular weakness and atrophy, which starts in the upper arms and lower legs and moves to the hips and shoulders, as well as a cardiac disease with conduction anomalies and arrhythmias [2].

Skeletal muscle weakness is a common symptom of all muscular dystrophies; however, the rate at which muscle weakening progresses differs depending on the type of muscular dystrophy. Additional clinical features seen in muscular dystrophies include cardiac abnormalities, joint contractures, respiratory impairments, postural deviations, and abnormal gait patterns [8].

Primary complications in pre-symptomatic stages of muscular dystrophy are delayed attainment of motor milestones and primarily delayed standing and walking. In the early ambulatory phase, the patient complains of difficulty in rising from a supine position along with calf enlargement. In this phase, the patient also faces difficulty in activities of daily living (ADL) such as running, jumping, and climbing stairs. Breathing is compromised due to scoliotic posture and respiratory muscle weakness. The late ambulatory phase is characterized by kyphoscoliosis, severe upper limb scoliosis, cardiomyopathy, and contractures. Other complications include varied intellectual performance and osteoporosis [12].

Physiotherapy in activities of daily living (ADL)

As the disease progresses, the clinical symptoms become more complicated, resulting in a growing loss of ability to perform daily activities (ADL). The life expectancy of individuals with muscular dystrophy has grown in response to advancements in the rehabilitative program, which includes respiratory and cardiovascular treatment. As a result, one of the most essential goals of patient treatment is to maintain ADL. Patients' deficits are assessed using the functional independence measure (FIM), a validated functional evaluation, with movement being the simplest to assess, followed by bowel control and feeding. This is because many patients were able to maintain their mobility as they grew older with the help of a customized electrical wheelchair with a seating system and a non-invasive ventilator. DMD is known to cause problems with language impairment and attention complexity [13]. Therefore, it is necessary to pay attention to the gross motor and to the cognitive functions while assisting a patient with muscular dystrophy [14].

Physiotherapy in gross motor functions

In muscular dystrophy, gross motor functions can be evaluated using the Bruininks-Oseretsky Test of Motor Proficiency-Short Form (BOTMP-SF) [15]. Difficulty in climbing stairs, running, and walking; the loss of ambulation; and history of falls are notable symptoms as the disease progresses further in nature. Problems in coordination and balance are also present. One of the largely affected aspects was the bilateral coordination between the lower and upper extremities, which is a part of gross motor proficiency [16].

Physiotherapy in preventing complications

As stated earlier, muscular dystrophies are progressive and lethal neuromuscular disorders. The goal of treatment is to improve the patient's quality of life by controlling symptoms and preventing further deterioration of his or her current condition [17]. Since there is no definitive cure for muscular dystrophies, the rehabilitation program should be customized concerning the patients' functional status: posture, quality of life, function, joint contractures, and participation in activities of daily living [18]. Musculoskeletal care is to preserve the posture, maintain a straight spine, reduce joint contractures, maintain motor function, and contribute to better bone health. Early therapy of joint contractures at the onset of the disease reduces the need for interventions and aids in the maintenance of posture and motor functioning. The worsening of contractures is prevented by approaches such as splinting, stretching, and serial casting. Due to poor mobility along with progressive muscle weakness, neuromuscular scoliosis develops in these patients. Early management of the same is required to preserve the functionality, facilitate activities of daily living, and most importantly alleviate pain. Scoliosis causes activity restrictions, respiratory and cardiac difficulties, seizure thresholds, ambulation, and back and rib pain, among other things [17]. Gluco-corticosteroid treatment along with rehabilitation is a promising treatment that slows down disease progression and also prolongs ambulation, maintains respiratory function, prevents further deterioration, and prevents and reduces the prevalence of serious deformity. Contracture management (stretching, positioning, standing frames, and standers), orthotic interventions, strength and endurance exercises, postural re-education, respiratory management, mobility, and upper limb support devices are all examples of interventions that can be used to improve function and participation [18].

Cardiomegaly is the most common cardiology problem in all types of muscular dystrophy. A dystrophin shortage in the heart causes it, and it is the major cause of death and morbidity in patients with muscular dystrophy [19]. Respiratory problems are the leading cause of morbidity and mortality in DMD patients [19]. Monitoring respiratory muscle performance and the timely application of lung volume recruitment, supported coughing, nocturnally assisted ventilation, and daytime ventilation are all examples of an anticipatory approach to therapy [19]. Any therapy approach for patients with MD must include a regular assessment of cardiopulmonary function [17].

Recent situation challenges

Given the COVID-19 pandemic restrictions, the healthcare system was forced to remodel itself. Due to the prevailing situations and social distancing protocols, rehabilitation was difficult to conduct on a physical basis. Many therapy services have been discontinued as a result of the closures of schools and outpatient therapy institutions caused by the COVID-19 crisis [20]. There was a need for telerehabilitation protocol. The World Confederation for Physical Therapy (WCPT) and the International Network of Physiotherapy Regulatory Authorities (INPTRA) in 2020 published a report that concerned digital physical therapy [21]. Online rehabilitation workshops were the need of the prevailing situation. Patients with muscular dystrophy show a decline in health status when a continuous rehabilitative protocol is not maintained [22]. Therefore, about COVID-19 protocols, rehabilitation could be conducted via online mode for the patients suffering from muscular dystrophy. The workshops can be conducted via various mediums of online meeting platforms. The workshops could be pre-recorded or live, following social distancing protocols. The physiotherapist can present all the exercises using a manikin, and the patients should be made to follow the therapist. Owing to this fact, the videos should be pre-recorded, or if there are daily live sessions, then every live session should be recorded and uploaded to any platform that is easily accessible anytime. One drawback of these online or pre-recorded sessions is that the families coming from a very poor socio-economic background cannot afford a smartphone or any device that could access these videos. For such patients, an offline workshop can be conducted, and the caregivers can be taught about the rehabilitation protocols, and a home program can be framed [18].

Home exercise program

Under the supervision of physical and occupational therapists, a daily prophylactic home stretching program should be started before the loss of passive ranges of motion. Stretching is recommended in regions where contracture or deformity is a concern. Stretching of the ankles, knees, and hips should begin shortly after diagnosis and continue throughout maturity [23].

## Review

Purpose

To prevent complications, continuous physiotherapy is advised for children with DMD, which is even asked to perform at home as a home program. Thus, the home program plays an important role in maintaining the health status of children with DMD. In the present pandemic scenario, the treatment for these children has been profusely affected. Even the home program was affected to the same level. There is a need to understand how children are getting affected and the way the home program is being given.

Objective

Our present scoping review aims to identify the present home program patterns being followed for children with DMD and their scope for recovery during the COVID-19 scenario.

Materials and methodology

Using the Preferred Reporting Items for Systematic Reviews and Meta-analyses extension for Scoping Reviews (PRISMA-ScR), we developed a search protocol [24]. This scoping review provides the answer to a specific question, i.e., the changing trends in the role of a home exercise program for patients with muscular dystrophy. To date, there has been no such scoping review of this topic because of the promotion of health at home; the concept came into the trend with the COVID-19 pandemic. Since then, this relatively new concept became a boon for MD patients.

The data were collected from electronic databases including PubMed, ProQuest, Cochrane, and Web of Science using the five-stage review framework proposed by Arksey and O'Malley [25]. We searched four electronic databases until June 2021. We included the published case studies, observational studies, and some systematic reviews that described the positive impacts of home exercise programs and the complication prevention with physiotherapy. We used the following keywords (combined using the Boolean operators "AND" and "OR") to test our electronic search approach for retrieving relevant studies: muscular dystrophy, home exercise program, COVID-19, physiotherapy, and rehabilitation.

Each title and/or abstract of studies generated using the search technique was independently examined by two review authors to identify the research that may be eligible. Studies were retrieved and independently assessed for eligibility. Any differences between the three independent assessors will be discussed with the third investigator.

Eligibility criteria

**Figure 1:**
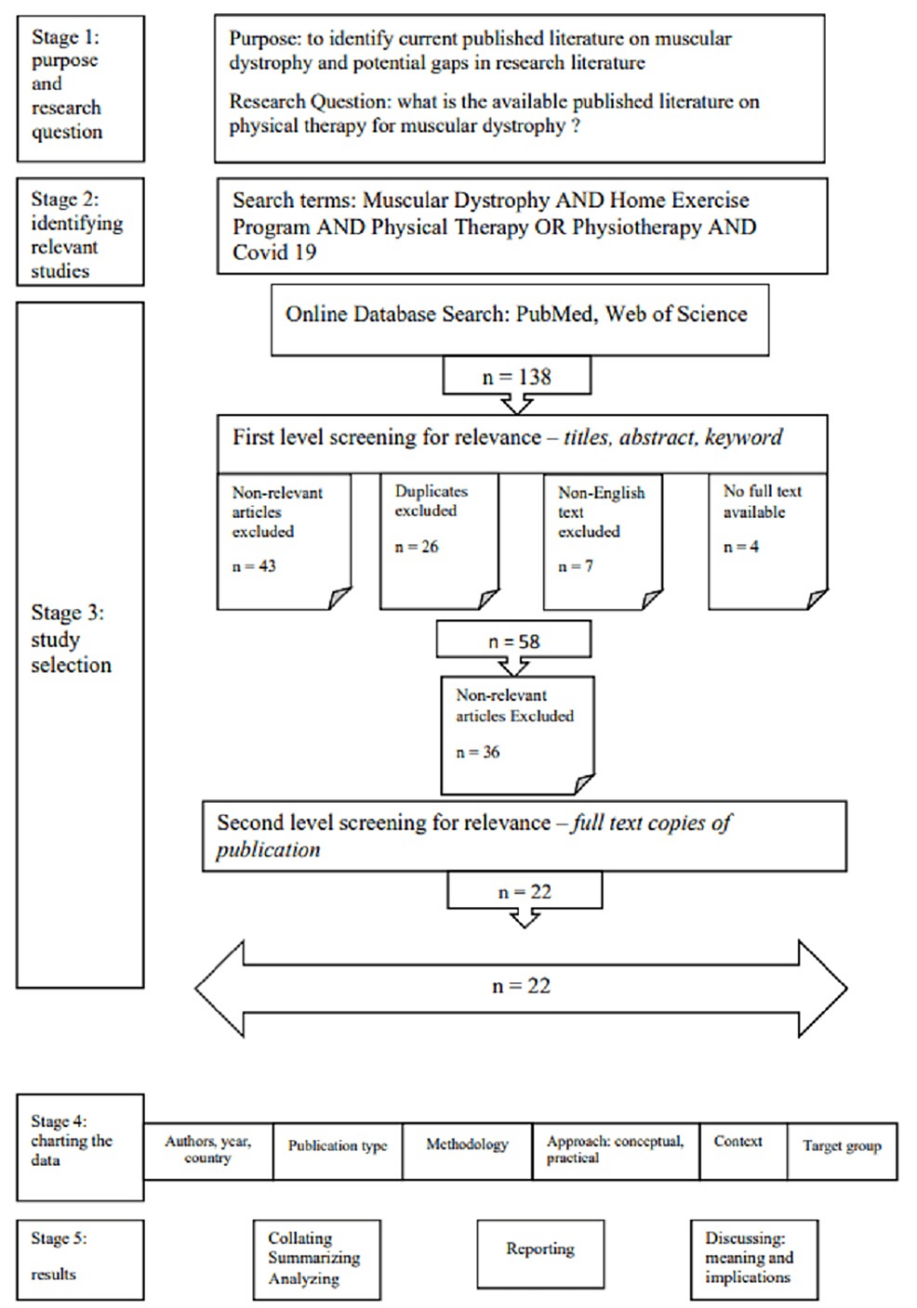
Flowchart of the study

Studies that match the following criteria will be considered: 1) population: patients with any type of muscular dystrophy; 2) study setting: assessments taking place at a hospital, a laboratory, or any rehabilitation center; and 3) study type: randomized controlled trials and observational studies. The search will be limited to human studies that have been published in peer-reviewed journals in full-text English articles. Figure 1 provides further details.

The proposed scoping review is conducted under Joanna Briggs Institute (JBI) methodology [26] for scoping reviews and according to the Preferred Reporting Items for Systematic Reviews and Meta-analyses extension for Scoping Reviews (PRISMA-ScR) [24].

Results

One hundred thirty-eight titles were screened, and 58 met the inclusion criteria. Most of them were observational studies and systematic reviews. Along with regular physiotherapy, the incorporation of HEP helped in early complication prevention in patients with muscular dystrophy. The HEP was found to be a successful adjunct in the COVID-19 scenario. The articles are provided in Table 1.

**Table 1:** Matrix of the articles selected for review

S. no.	Title	Author, year	Journal	Study design	Main finding
1	Establishing a telerehabilitation program for patients with Duchenne muscular dystrophy in the COVID-19 pandemic	Sobierajska-Rek et al., 2021 [18]	Springer Nature	Original article	Patients can continue home-based therapy with the support of carers and physiotherapist's direction (online communication or video). Parents and carers of Duchenne muscular dystrophy (DMD) patients prefer online videos/instructions/video guidance to live workshops.
2	The care of patients with Duchenne, Becker, and other muscular dystrophies in the COVID‐19 pandemic	Veerapandiyan et al., 2020 [20]	Muscle & Nerve	Review article	They stress the need of patient, family, and healthcare provider collaboration when making treatment decisions, taking into account any COVID-19-specific restrictions and precautions.
3	Respiratory telerehabilitation of boys and young men with Duchenne muscular dystrophy in the COVID-19 pandemic	Sobierajska-Rek et al., 2019 [27]	International Journal of Environmental Research and Public Health	Observational study	The findings demonstrate that respiratory telerehabilitation can be used in DMD patients; however, carers should be involved.
4	Motor activity and Becker's muscular dystrophy: lights and shadows	Lanza et al., 2020 [28]	The Physician and Sportsmedicine	Review	Mild-to-moderate intensity aerobic exercise tailored to the patient's needs should be explored as part of their treatment.
5	Current and emerging therapies in Becker muscular dystrophy (BMD)	Angelini et al., 2019 [29]	Acta Myologica	Review	Multidisciplinary treatment with physiotherapy is used to minimize joint contractures and increase walking distance. To adjust treatment to particular cases, personalized medicine is essential.
6	Activities of daily living (ADL) structure of patients with Duchenne muscular dystrophy, including adults	Fujiwara et al., 2017 [14]	The Keio Journal of Medicine	Cross-sectional survey	Patients with DMD have a distinct difficulty with their ADL. The proportional level of independence/dependence determines the order of difficulty.
7	Duchenne muscular dystrophy: a practice update	Suthar and Sankhyan, 2018 [12]	Indian Journal of Pediatrics	Review article	A boy with DMD can improve ambulation, independence, and quality of life and delay disease-related consequences with effective care.

Discussion

This scoping review presents the literature on muscular dystrophy and the role of physiotherapy and home exercise programs in the COVID-19 scenario. Some studies present the importance of HEP for different diseases; this study shows how HEP is helpful for patients with MD and how it improves the overall quality of life and improves the activities of daily living (ADL) and gross functions, preventing complications.

As per Sobierajska-Rek et al., patients can continue home-based therapy with the support of caregivers and physiotherapist's direction (online communication or video) [18]. According to Veerapandiyan et al., a multidisciplinary approach with a teamwork of different departments along with the involvement of caregiver will provide a newer way of understanding and applying therapy to patients with DMD [20].

 Angelini et al. in a review said that to reduce joint contractures and increase walking distance, multidisciplinary treatment including physiotherapy is performed. Personalized medicine is required to tailor treatment to specific cases [29]. Similarly, in a cross-sectional survey by Fujiwara et al., the maintenance of ADL and the formulation of a program to avoid disability advancement, as well as supportive counseling for patients and family, are the main goals of rehabilitation for patients with DMD. As a result, it's critical to understand the level of difficulty connected with each ADL, as well as how ADL lowers with age [14].

There are studies on cerebral palsy (CP) where the mother was used as a rehabilitative aid, and there was a tremendous improvement in the performance of the children with CP [30]. This opens up a window for similar studies to be done in the patients with muscular dystrophies as in present time, children spend the whole day with mothers at their home.

## Conclusions

This review presents different therapeutic measures that can be taken for the prevention of complications in patients with muscular dystrophy. It also takes into account how the home exercise program plays an important role in removing the gaps. The advantages of the home exercise program in the COVID-19 scenario were also demonstrated in this review. Be it at home or in a rehabilitation facility, constant practice is crucial in achieving success and avoiding complications. While rehabilitating a child with muscular dystrophy, a home exercise program should also be provided as it enhances the recovery process.
